# Nighttime activities and peripheral clock oscillations depend on *Wolbachia* endosymbionts in flies

**DOI:** 10.1038/s41598-018-33522-8

**Published:** 2018-10-18

**Authors:** Eri Morioka, Minami Oida, Tsutomu Tsuchida, Masayuki Ikeda

**Affiliations:** Graduate School of Science and Engineering, University of Toyama, 3190 Gofuku, Toyama, 930-8555 Japan

## Abstract

*Wolbachia* are ubiquitous bacterial endosymbionts of arthropods and affect host gene expression. Although *Wolbachia* infections were suggested to modulate sleep in flies, their influence on the circadian clock remained obscure. Here, we screened bacterial symbionts in a laboratory *Drosophila melanogaster* colony, and observed widespread infections of wMel strain *Wolbachia*. We established a *Wolbachia*-free strain from a clock gene reporter strain, *period-luciferase* (*per-luc*). Temperature (19–29 °C)-compensated free-running periods were detected regardless of infections which may reflect the lack of wMel infections in central circadian pacemaker neurons. However, locomotor activity levels during the night or subjective night were significantly amplified in uninfected flies. Moreover, the behavioral phenotype of F1 offspring of an uninfected female and infected male resembled that of uninfected flies. This trait is consistent with maternal transmission of *Wolbachia* infection. Interestingly, *per-luc* activities in headless bodies, as an index of peripheral circadian oscillators, were severely damped in uninfected flies. Additionally, circadian amplitudes of PER immunoreactivities in Malpighian tubules were reduced in uninfected flies. These results demonstrate that *Wolbachia* boost fly peripheral clock oscillations and diurnal behavioral patterns. Genetic mechanisms underlying behavioral rhythms have been widely analyzed using mutant flies whereas screening of *Wolbachia* will be necessary for future studies.

## Introduction

Intracellular alpha proteobacterium *Wolbachia* are ubiquitous endosymbionts of arthropods^1–3^. They are maternally transmitted to offspring and are involved in sex determination^4^, sperm–egg cytoplasmic incompatibility^5^, and pathogen defense^6,7^. *Wolbachia* infections were also recently suggested to increase nighttime sleep in flies by transcriptionally regulating dopamine synthetic enzymes^8^. However, despite their wide range of influences on host activities, the cellular mechanisms underlying *Wolbachia*-mediated sleep regulation are not fully understood.

The involvement of symbiosis in host circadian activities has been demonstrated in several species. For example, symbiosis between the squid *Euprymna scolopes* and the luminous bacterium *Vibrio fischeri* enables the daytime switch-off of light-emitting organs^9^. Interestingly, synchronous gene expression patterns were found for these symbiotic partners over the course of a day–night cycle, and metabolic modes were switched between day and night in the organ. Additionally, the notable influence of endosymbionts on host circadian rhythms has been reported in the unicellular ciliate *Paramecium bursaria*^10,11^. Its symbiotic algae, *Chlorella*, enable the host’s mating reactivity rhythm to persist under constant light conditions, as shown by the fact that *Chlorella*-free cells derived from the same strain fail to display such a rhythm. However, whether such a dynamic influence of symbiosis on host circadian activities could be the general case in many animal species is currently unknown.

Daily temporal patterns from cellular activities to animal behaviors are largely governed by the endogenous circadian clock system, which has been proposed to be driven by gene transcription–translation feedback loops. Circadian oscillations in “clock genes” have been observed in a wide range of systems, from unicellular organisms to mammalian cells, and from peripheral organs to the central nervous system^12–14^. It is widely accepted that the circadian clock system in animals consists of central pacemaker neurons and various peripheral oscillator cells. For example, in mammals, circadian rhythms in PER-immunoreactivity (ir) and/or *per* promoter-driven luciferase (*per-luc*) levels were observed not only in central pacemakers (i.e., hypothalamic suprachiasmatic nucleus neurons) but also in fibroblasts^15^, livers, lungs, and muscles^13^. Additionally, circadian rhythms in fly PER-ir and/or *per-luc* levels were reported not only in central pacemaker neurons^16–18^ but also in Malphigian tubules (MTs)^19^, prothoracic gland cells^20–22^, antenna, legs, and wings^23,24^. Although *Wolbachia* affect various host gene expression^8,25^, its effect on clock gene transcriptional rhythms are currently unknown.

We have found frequent infection by wMel strain *Wolbachia* in fly strains used for circadian rhythm assays and kept within enclosed laboratory conditions. Thus, we established a *Wolbachia*-free fly strain from *per-luc* strain flies using tetracycline to ensure freedom from infection. It is generally accepted that the circadian clock provides three major functions: (i) self-sustaining oscillations, (ii) temperature-compensated oscillations (i.e., stability against temperature perturbations), and (iii) ability to entrain to environmental time cues (i.e., temporal adjustment). Therefore, in the present study, we analyzed free-running locomotor activity rhythms under constant darkness (DD) at different ambient temperature levels and entrainment of rhythms to 8-h shifts of 12:12-h light/dark (LD) cycles in infected and uninfected *per-luc* flies. We also analyzed effects of *Wolbachia* symbiosis on PER-ir and *per-luc* levels in these flies to further understand the involvement of *Wolbachia* in molecular clock movements.

## Results

### Detection of endosymbionts and their elimination

We examined symbiotic bacteria in the *per-luc* line of *D. melanogaster* by analyzing 16S ribosomal RNA (16S rRNA) gene sequences amplified with PCR using the universal eubacterial primers. The restriction fragment length polymorphism (RFLP) analysis detected only one sequence type, which indicated the presence of one bacterial symbiont in the *per-luc* line. The sequence of the bacterial gene showed high similarity to 16S rRNA gene sequences of *Wolbachia* from various insects (Fig. S1A). Detailed analysis using the *Wolbachia* surface protein (*wsp*) gene was conducted to clarify the phylogenetic position of the *Wolbachia* species. RFLP and sequence analysis of the *wsp* gene showed that the *per-luc* line contained only one *wsp* sequence. Molecular phylogenetic analysis of the *wsp* sequence revealed that the symbiont of the *per-luc* line belonged to the wMel group of *Wolbachia* (Fig. S1B). All individuals of the *per-luc* line were infected with wMel (24/24 individuals).

To examine the effects of *Wolbachia* infection on host circadian rhythms, we generated *Wolbachia*-free *per-luc* flies. Following antibiotic treatment (50 µg/mL tetracycline in fly foods) for three generations, a single female was transferred to an isolation vial to establish isofemale lines. Uninfected offspring were selected and supplied with tetracycline-free regular food. Elimination of *Wolbachia* was confirmed by PCR (Fig. S2) or by immunohistochemistry against a heat shock protein (hsp60)^26–28^.

Body weight was compared among infected and uninfected adult male (0.67 ± 0.01 mg for infected flies and 0.66 ± 0.01 mg for uninfected flies, N = 10 for both groups) and female flies (1.12 ± 0.02 mg for infected flies and 1.10 ± 0.01 mg for uninfected flies, N = 10 for both groups). No significant difference in body weight was found between infected and uninfected groups (n.s. by Student’s *t*-test irrespective of sex).

### The effects of *Wolbachia* endosymbionts on host behavioral rhythms

**Figure 1:**
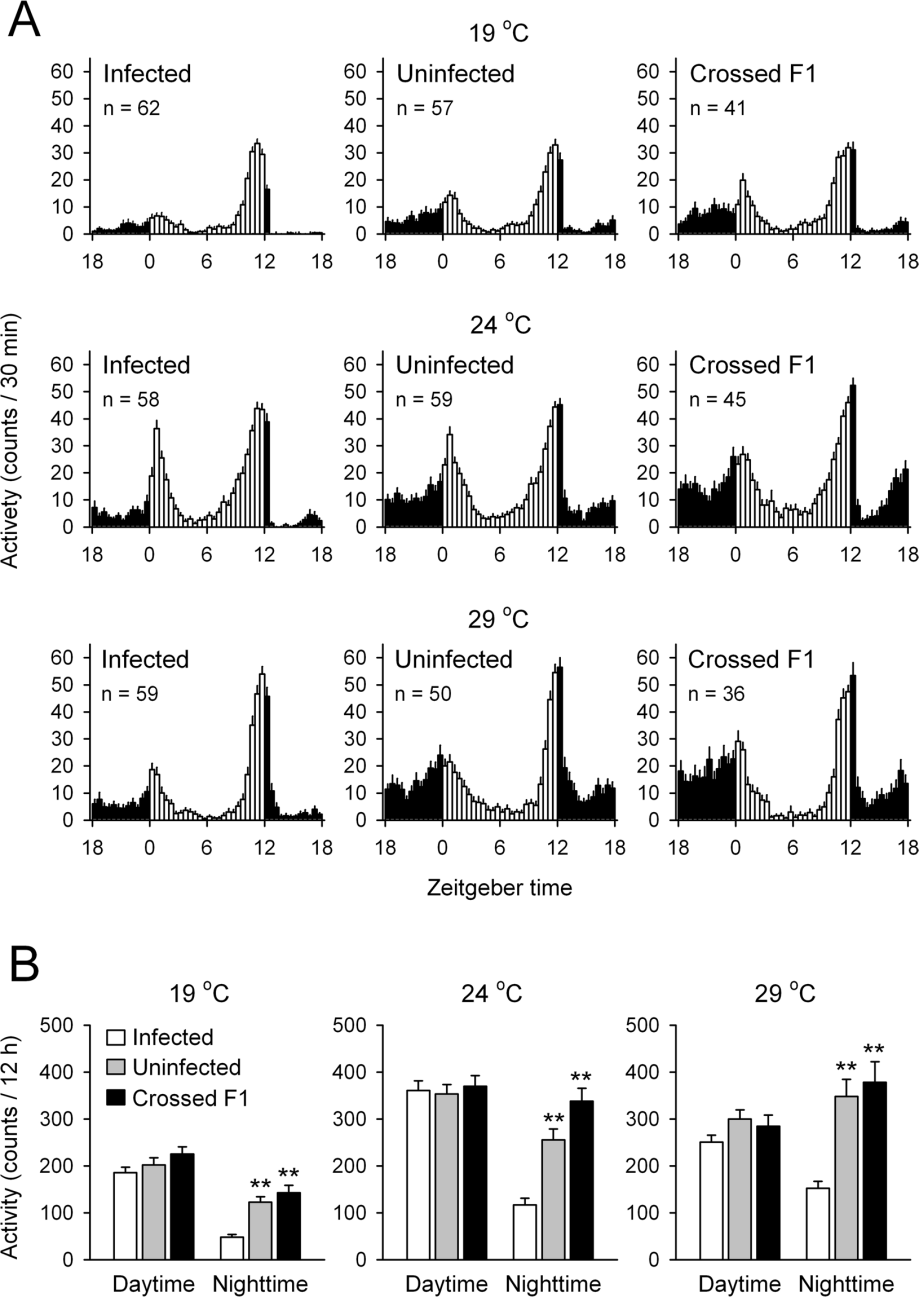
Locomotor activity rhythms of infected, uninfected, and crossed F1 *per-luc* flies kept under LD cycles at various ambient temperatures. (**A**) Histograms show mean activity for each 30 min at various ambient temperatures (19, 24, and 29 °C). White and black bars indicate day and night, respectively. (**B**) Total activity counts of infected, uninfected, and crossed F1 flies during the daytime (ZT0-12; white columns) and nighttime (ZT12-24; black columns) under LD cycles. Data are presented as mean ± SE. *p < 0.05, **p < 0.01 compared with corresponding infected fly groups by one-way ANOVA followed by Duncan’s multiple range test. n, number of flies analyzed.

We analyzed locomotor activity in infected and uninfected flies at different ambient temperatures (19, 24, and 29 °C). Under 12:12-h LD cycles, both infected and uninfected flies showed typical bimodal activity patterns consisting of morning and evening peaks regardless of temperature levels (Fig. 1A,B). Interestingly, total activity counts during the nighttime in uninfected strain flies were significantly higher than in infected flies at any temperature examined (Fig. 1B). To exclude the possible involvement of other microorganisms in the regulation of nighttime activities, we further analyzed locomotor activities in F1 offspring of uninfected females and infected males. Consistent with uninfected strain flies, crossed F1 flies showed a significant increase in total activity counts during the night (Fig. 1A,B).

**Figure 2:**
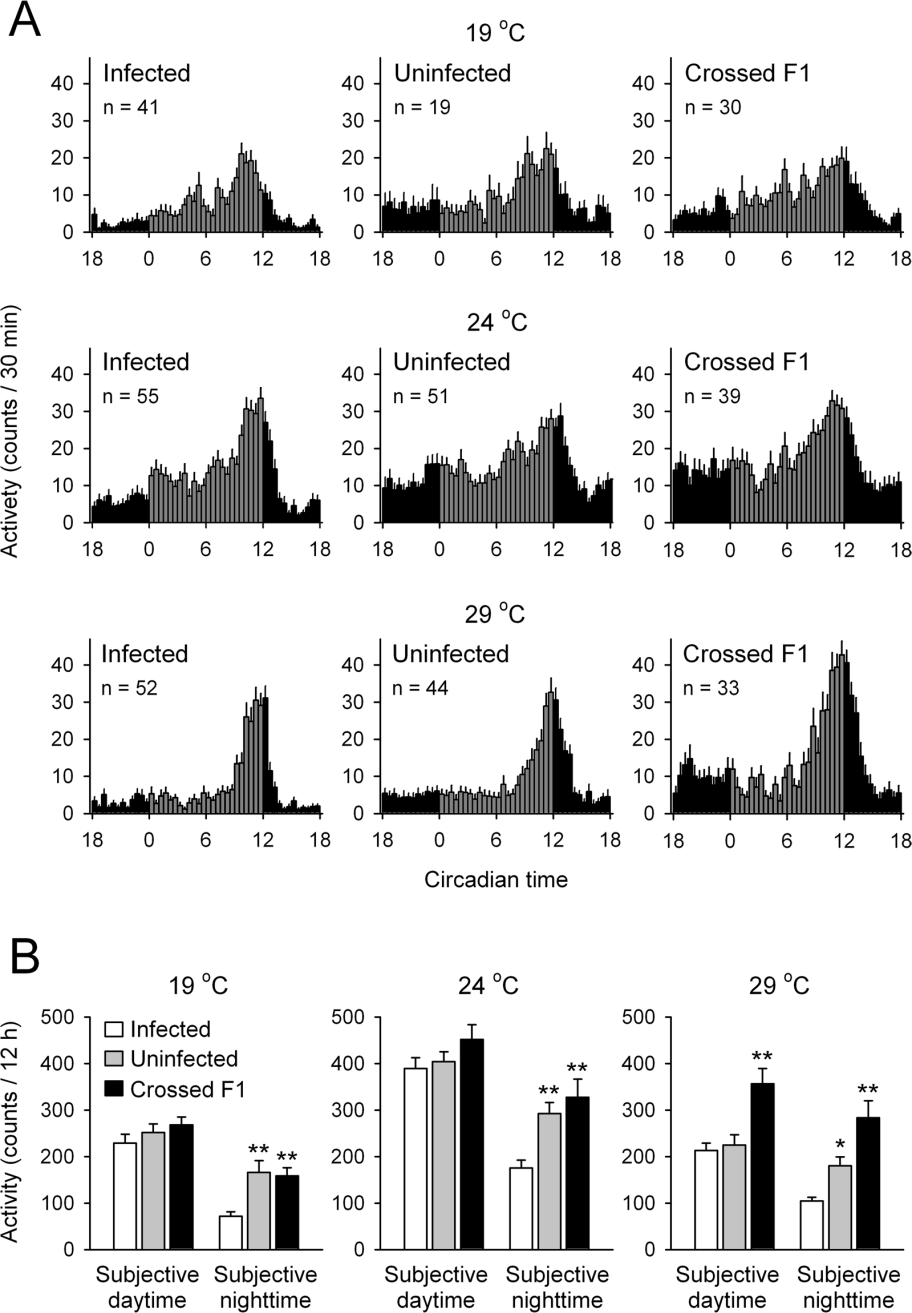
Locomotor activity rhythms of infected, uninfected, and crossed F1 *per-luc* flies kept under DD at various ambient temperatures. (**A**) Average locomotor activity was analyzed on the 4th day under DD conditions at 19, 24, and 29 °C. Histograms show mean activity for each 30 min. (**B**) Total activity counts of infected, uninfected, and crossed F1 flies during the subjective daytime (grey columns) and subjective nighttime (black columns) on the 4th day under DD conditions. Data are presented as mean ± SE. *p < 0.05, **p < 0.01 compared with corresponding infected fly groups by one-way ANOVA followed by Duncan’s multiple range test. n, number of flies analyzed. Details for circadian rhythm profiles are shown in Table 1.

Under DD, infected-, uninfected- and crossed F1 flies displayed temperature-compensated free-running rhythms; the average τs converged between 23.65 h and 23.94 h (Table 1; *Q*_10_ = 1.001 for infected group, 1.006 for uninfected group, and 1.008 for F1 group). No differences were found in the average τs in DD conditions at any temperature examined (Table 1). At 19 °C, a lower percentage of rhythmic flies occurred because of low temperature immobilization (Table 1, Fig. 2), yet total activity counts during the subjective nighttime were significantly higher in uninfected flies and crossed F1 flies (Fig. 2).

**Table 1:** Free-running locomotor activity rhythms of infected, uninfected and crossed F1 *per-luc* flies at different ambient temperatures.

	n	%R	Period (h)	Power	Activity
19 °C					
Infected	62	66.18 ± 5.32	23.79 ± 0.05	445.10 ± 16.43	1.20 ± 0.07
Uninfected	57	30.14 ± 5.06	23.65 ± 0.05	464.24 ± 22.73	1.37 ± 0.11
Crossed F1	41	71.96 ± 11.02	23.74 ± 0.05	443.52 ± 18.92	1.56 ± 0.10
24 °C					
Infected	58	94.58 ± 2.45	23.87 ± 0.04	623.69 ± 28.48	2.03 ± 0.13
Uninfected	59	84.81 ± 4.24	23.75 ± 0.04	497.91 ± 17.25	2.55 ± 0.16
Crossed F1	45	86.49 ± 3.99	23.84 ± 0.03	473.67 ± 17.53	3.11 ± 0.25
29 °C					
Infected	59	90.74 ± 2.29	23.83 ± 0.04	582.08 ± 22.82	1.14 ± 0.07
Uninfected	50	89.61 ± 6.56	23.80 ± 0.05	541.77 ± 30.15	1.52 ± 0.12
Crossed F1	36	93.08 ± 4.71	23.94 ± 0.05	585.40 ± 30.52	2.33 ± 0.21

n, number of flies analyzed; %R, percentage of rhythmic flies; Activity, activity counts per 6 min. See Figs 1 and 2 for activity levels.

**Figure 3:**
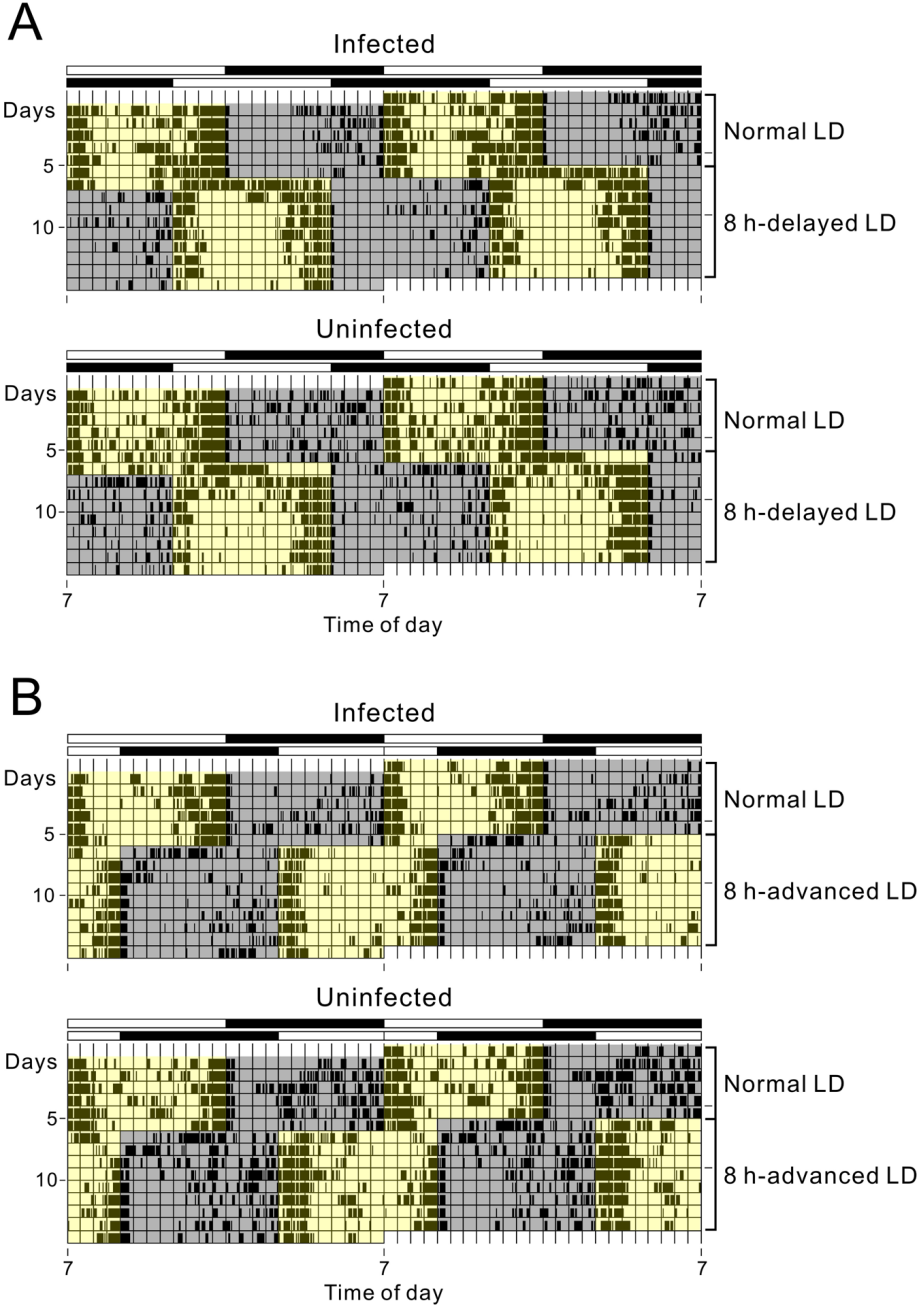
Representative double-plotted locomotor activity rhythms of infected and uninfected *per-luc* flies. Flies were kept under 12:12-h LD cycles at 24 °C and then subjected to an 8 h phase delay (**A**) or advance (**B**). The gray and yellow areas in the actograms indicate the dark and light phase, respectively. Bars on the top indicate LD conditions.

To investigate the effects of *Wolbachia* infection on photic entrainment of locomotor activity rhythms, infected- and uninfected flies were exposed to an 8-h advance or delay of LD cycles. Both groups of flies adjusted their circadian activity pattern immediately to the new LD regime (Figs 3, S3). Thus, *Wolbachia* infection seems to have little effect on photic entrainability of the circadian clock.

### Localization of *Wolbachia* endosymbiont

**Figure 4:**
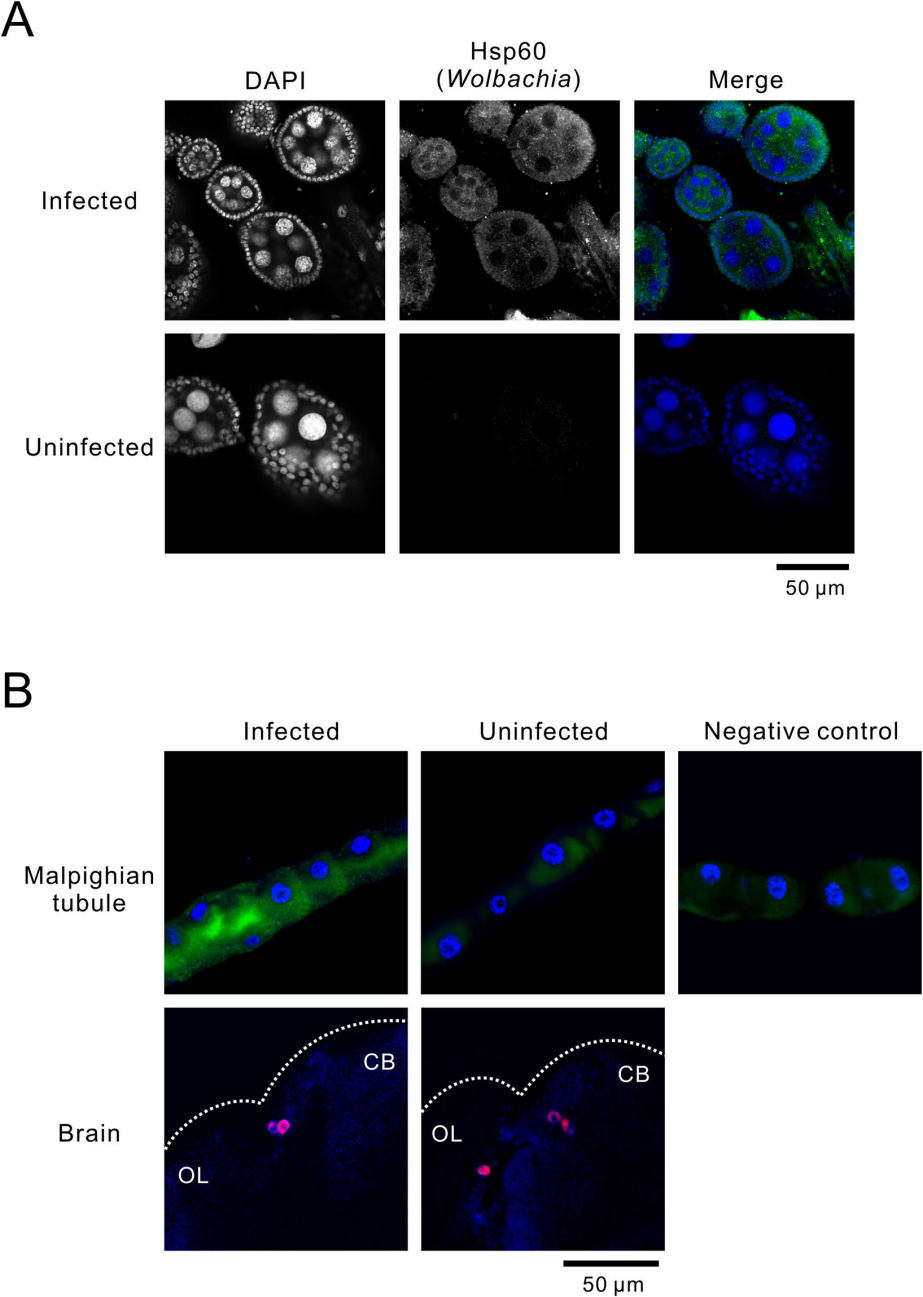
Localization of *Wolbachia* infection in fly organs. (**A**) Representative images of immunohistochemical identification of *Wolbachia* (green) in oocytes using an anti-heat-shock protein (hsp) 60 antibody. Host DNA is counter-stained with 4′,6-diamidino-2-phenylindole (DAPI; blue). (**B**) The same analysis of *Wolbachia* demonstrated high infection levels in Malpighian tubules but not in the brain. Small background green fluorescence was observed in the Malpighian tubules of uninfected flies. The background levels were equivalent to the staining results of infected Malpighian tubules without primary antibody (negative control). For brain staining, pigment-dispersing factor (PDF) was also stained as a marker for central circadian pacemakers (magenta). The approximate brain shape (dotted line) is determined by blue autofluorescence. CB, central brain; OL, optic lobe. Note that large LN_v_s were visualized in these frames.

To determine the localization of *Wolbachia* endosymbionts in flies, we stained oocytes, MTs, and brains with an anti-hsp60 antibody which labels *Wolbachia*^26–28^. In infected flies, anti-hsp60 signals were extensively present in oocytes (Fig. 4A) and various peripheral tissues of the adult body, including MTs, whereas signals were undetectable in the brain (Fig. 4B). Circadian pacemaker neurons (ventral lateral neurons; LN_v_s) in flies were labeled by an antibody against the pigment-dispersing factor (PDF)^29^, whereas hsp60 signals were lacking in these neurons (Fig. 4B). Relatively strong green fluorescent background levels were detected in all MTs but strong punctate signals were detected only in infected flies (Fig. 4B).

Absence of *Wolbachia* was further confirmed in physically isolated brains using real-time RT-PCR with the *wsp* promotor, where *wsp* transcriptions were undetectable in the brain homogenates regardless of *Wolbachia* infection status (Fig. S2B).

### The effects of *Wolbachia* endosymbionts on peripheral circadian clocks

**Figure 5:**
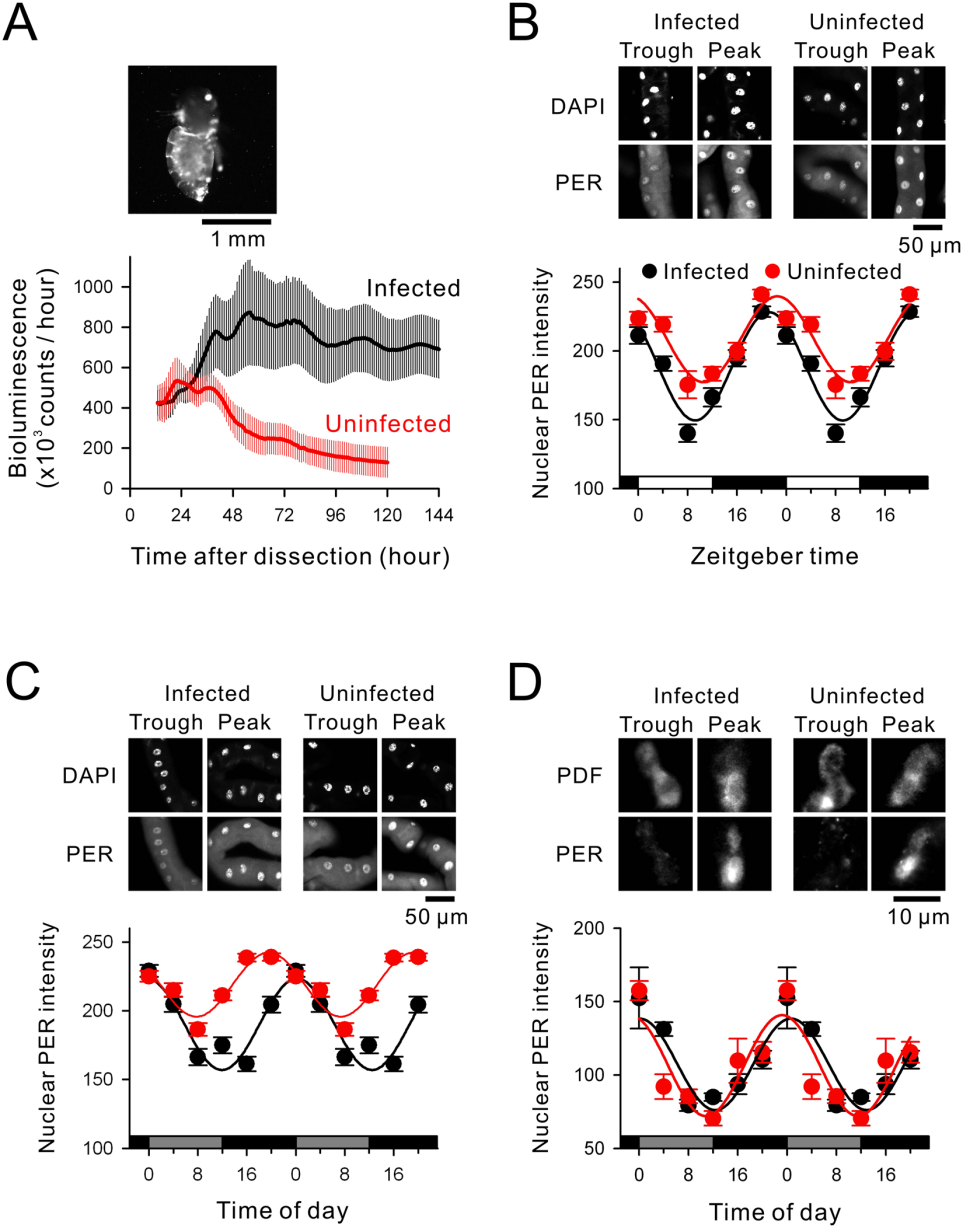
Clock gene (*per*) transcriptional/translational rhythms in infected (black) and uninfected (red) flies. (**A**) Average *per-luc* signals in headless fly bodies under DD conditions. Uninfected fly bodies exhibited damped *per-luc* oscillations. The *per-luc* bioluminescent image immediately before the chemiluminescent recording is shown on the top. (**B**–**D**) Nuclear PER intensity of intact flies kept under LD or DD conditions. Time-course changes in the immunofluorescent intensity of nuclear PER are double-plotted. (**B**) Nuclear PER intensity in Malpighian tubules of flies kept under LD. Representative PER immunostaining images are shown on the top. (**C**) The same analysis as (**B**) with flies on the 4th day under DD conditions. Note that the circadian amplitude of the PER expression rhythm was 30% smaller in uninfected Malpighian tubules under DD. (**D**) Nuclear PER expression in the central pacemaker neurons (s-LN_v_s) of flies on the 4th day under DD. Effects of *Wolbachia* infections were less evident for the PER expression rhythms of s-LN_v_s.

Based on the histological localization of *Wolbachia* endosymbionts, *per-luc* bioluminescence was compared in headless fly bodies. In infected flies, the bioluminescent intensity displayed circadian oscillations over five cycles (Fig. 5A, mean bioluminescence counts per hour = 702.6 × 10^3^; mean period = 23.73 ± 1.57 h; N = 4). However, uninfected flies exhibited severely damped oscillations with circadian oscillations only detectable for 2–3 cycles (mean bioluminescence counts per hour = 294.0 × 10^3^; mean period = 23.57 ± 3.00 h; N = 4) (Fig. 5A).

To further examine cellular circadian rhythms in intact flies during different lighting conditions, we analyzed nuclear PER-ir in central circadian pacemaker neurons (small LN_v_s; s-LN_v_s) and typical peripheral clock cells (MTs). In MTs under LD conditions, the circadian amplitude of PER-ir rhythms in uninfected flies was 20.7% smaller than in control flies (Fig. 5B, number of tissues = 85; number of cells = 671). Also in MTs in DD conditions, the circadian amplitude of PER-ir rhythms in uninfected flies was 30% smaller than in control flies (Fig. 5C, number of tissues = 39; number of cells = 834). However, PER-ir rhythms in s-LN_v_s was rather partially (10.4%) increased in uninfected flies (Fig. 5D, number of tissues = 54; number of cells = 137).

## Discussion

*Wolbachia* are intracellular alpha proteobacteria that are maternally transmitted to offspring and are widespread in arthropods^1–3^. Our laboratory *Drosophila* colony is composed of multiple fly strains from all over the world, including those from Bloomington Drosophila Stock Center (Indiana University, USA), and the Vienna Drosophila Resource Center (Austria). Approximately half of the clock mutant fly stains randomly tested in our laboratory colony were infected with *Wolbachia* (Fig. S2A). The presence of *Wolbachia* in laboratory fly colonies has also been reported by other groups^30–32^. These frequent infections are not surprising because more than 60% of insect species, including *D. melanogaster*, are infected by *Wolbachia*^2^. Thus, we speculate that many *D. melanogaster* studies, even those performed using in a closed laboratory environment, are under the influence of *Wolbachia* symbionts.

The most dramatic behavioral phenotype observed in this study was a significant increase in nighttime activities in uninfected flies regardless of ambient temperature levels. At 29 °C under LD cycles, average nighttime activities were beyond the daytime levels, resulting in a loss of diurnal locomotor rhythms in these flies. To determine the specific influence of *Wolbachia* symbionts, we analyzed locomotor activities in F1 offspring of uninfected females and infected males. Because most of the other microorganisms including the gut-associated microenvironment are restored in the F1 offspring^33^, we compared the phenotypes with those of fully sterilized flies. The observed increased nighttime activities in F1 offspring were similar to those seen in uninfected strain flies, suggesting the importance of *Wolbachia* symbionts for the manifestation of apparent diurnal rhythms in flies. To further explore these mechanisms, we analyzed the temporal distribution of locomotor activities under DD, revealing increases in locomotor activities during subjective nighttime in uninfected strain flies and F1 offspring. Thus, the influence of *Wolbachia* on nighttime activities was not the result of “masking effects” which are not mediated by circadian clock oscillations.

Heterogeneity of peripheral circadian clocks has been reported in *D. melanogaster*^34^: endocrine oscillators in the prothoracic gland are strongly regulated by central pacemakers^22^ whereas MTs contain independent oscillators^19^. The present study demonstrated damped *per* transcriptional oscillations in the peripheral circadian clocks of uninfected flies. Moreover, hsp60 and PER immunostaining assays revealed that *Wolbachia* symbionts have little effect on central circadian pacemaker neurons, making unique unbalanced clock oscillations within uninfected fly bodies. Because free-running rhythms and photic entrainment of circadian rhythms are indistinguishable between infected- and uninfected flies, a reduction in peripheral clock oscillations may have limited effect on basic clock movements. However, we suggest that daily activity patterns in flies are under the strong influence of peripheral clock movements and *Wolbachia* symbionts.

*Wolbachia* infection has previously been documented in the *Drosophila* brain, but the extent of infection was dependent on the *Wolbachia* strain, host *Drosophila* strain, and brain region^35^. Indeed, the *Wolbachia* strains wRiv and wPop showed higher titers in the brain than wMel, while higher *Wolbachia* titers were seen in *D. simulans* than in *D. melanogaster*^35^. Correlation analyses suggested that *Wolbachia* infection influenced sleep time in flies^35^. Furthermore, Bi *et al*.^8^ recently reported that wMel infection increased nighttime sleep quantity under LD cycles in *D. melanogaster*. Thus, the present results of an increase in nocturnal activities in uninfected flies may be caused by the reflective effects of sleep disturbance. Bi *et al*.^8^ found that *Wolbachia* may affect dopaminergic activities because two essential genes involved in dopamine synthesis, *Pale* and *Ddc*, were significantly upregulated in *Wolbachia*-infected flies. In the present study, we also observed partial enhancement of PER-ir rhythms in s-LN_v_s in uninfected flies. However, wMel infections were barely detectable in the brain of *D. melanogaster*, including in PDF-positive pacemaker neurons. This result is consistent with the findings of Albertson *et al*.^35^. Thus, the actions of wMel on central neurons could be the result of indirect influences on peripheral systems.

The effect of *Wolbachia* symbionts may influence diverse host cellular activities. Of these, one of the most common phenotype induced by *Wolbachia* is sperm–egg cytoplasmic incompatibility (CI), a form of male-derived zygotic lethality^1,5,36^. Additionally, a series of studies demonstrated that *Wolbachia* infections elevated reactive oxygen species and caused changes in the redox homeostasis of host cells^37,38^. Furthermore, a microarray assay for filarial nematodes demonstrated that diverse genes regulating translation, transcription, protein folding/sorting, metabolic control, and intracellular signaling are under the control of *Wolbachia* symbionts^25^. The synthesis of heme in filarial nematodes is dependent on *Wolbachia*, so heme-dependent energy metabolisms, which can be compensated for by mitochondrial gene expression, are under the strong influence of *Wolbachia* symbionts^25^. This indicates that metabolic oscillators, as supportive components of clock gene transcription rhythms^39^, could also be under the influence of *Wolbachia* symbionts.

Oxidation and reduction cycles of peroxiredoxin represent a circadian periodicity in a diverse range of cells from human red blood cells to bacteria, so the redox cycle may be an evolutionarily conserved, nuclear-independent circadian oscillator^40–42^. Thus, we speculated that oxidation and reduction cycles may be primary candidates for *Wolbachia*-dependent circadian oscillators. Based on this hypothesis, we examined the effects of *Wolbachia* symbionts on the levels of oxidized peroxiredoxin in headless fly bodies. However, oxidation rhythms were observed both in infected and uninfected flies with shifts in their circadian phases (Fig. S4). Therefore, further studies are required to uncover the intracellular mechanisms regulating *Wolbachia*-dependent *per* oscillations in peripheral circadian clocks.

Here, we demonstrated that a ubiquitous *Wolbachia* endosymbiont could influence peripheral clock oscillations and circadian activity patterns in *Drosophila* models. Notably, in 2017, the US Environmental Protection Agency approved the utilization of *Wolbachia* to kill wild mosquitoes that transmit viruses such as dengue, yellow fever, and Zika^43^. Based on our findings, we suggest that future work with *Wolbachia*, including other *Wolbachia* strains, and other native endosymbionts should consider whether they modulate the circadian behavioral patterns of insect species. Such a question is beyond the scope of the current study but is an important concern.

## Methods

### Fly stocks

The *per-luc* (second chromosome) line of *D. melanogaster*, which was originally obtained from Dr. Jeffery C. Hall (Brandeis University, Waltham, MA) via Dr. Akira Matsumoto’s laboratory (Juntendo University, Tokyo, Japan), was predominantly used in this study. Three additional lines of *D. melanogaster* (*per*^*01*^, *Clk*^*Jrk*^, and *cry*^*01*^) were used to examine infection frequencies of *Wolbachia*. The *per*^*01*^ line was kindly provided by Dr. Akira Matsumoto’s laboratory. The *Clk*^*Jrk*^ and *cry*^*01*^ lines were kindly provided by Dr. Taishi Yoshii’s laboratory (Okayama University, Okayama, Japan). Flies were raised on cornmeal, molasses, and yeast food at 25 ± 1 °C under 12:12-h LD (light on at 07:00) cycle conditions.

### DNA extraction

DNA was extracted using a standard phenol: chloroform protocol. For symbiont screening, 10 individuals (five females and five males) of the *per-luc* line were pooled and homogenized with a sterile plastic pestle (Kimble Chase, Rockwood, TN) in 200 µL of STE buffer [100 mM NaCl, 10 mM Tris-HCl (pH 8.0), 1 mM EDTA (pH 8.0)]. For specific PCR detection of *Wolbachia*, DNA was extracted from one individual of the *per-luc* line. Purified DNA was dissolved in 400 µL (for the samples extracted from 10 pooled individuals) or 200 µL (for the samples extracted from one individual) of TE buffer [10 mM Tris-HCl (pH 8.0) and 0.1 mM EDTA].

### Detection of endosymbionts

16S rRNA genes of whole eubacteria from the whole-insect DNA extract were amplified by PCR with *Ex Taq* DNA polymerase (Takara Bio, Tokyo, Japan) using the forward primer 16SA1 (5′-AGAGTTTGATCMTGGCTCAG) in combination with the reverse primer 16SB1 (5′-TACGGYTACCTTGTTACGACTT) as described previously^44^. The protocol utilized a temperature profile of 94 °C for 2 min followed by 35 cycles of 94 °C for 30 sec, 55 °C for 30 sec and 72 °C for 1 min. The *wsp* gene was also amplified using the forward primer wspF (5′-GGGTCCAATAAGTGATGAAGAAAC) in combination with the reverse primer wspR (5′-TTAAAACGCTACTCCAGCTTCTGC)^45^ under the same temperature profiles. The PCR products were cloned, and 24 clones of each gene were subjected to RFLP genotyping using RsaI and HaeIII, before being sequenced as previously described^44^. The sequence similarity of a detected symbiont was analyzed by BLAST^46^ on nucleotide sequences deposited with DDBJ/NCBI/GenBank databases. Candidate taxa for phylogenetic analysis were selected from a variety of bacterial 16S rRNA gene or *wsp* gene sequences including those known for bacteria isolated from insects and those showing high similarity scores in a BLAST search. Phylogenetic analysis of the symbiont was conducted using the Neighbor-joining method in the program MEGA 5^47^. Bootstrap tests were conducted with 1,000 resamplings. Specific PCR was conducted to examine the infection frequency of *Wolbachia* in the *per-luc* line using the primers wspF and wspR.

### Nucleotide sequence accession numbers

The nucleotide sequences determined in this study were deposited in the DDBJ/EMBL/GenBank nucleotide sequence database under accession numbers LC108848 (*Wolbachia* 16S rRNA gene) and LC108849 (*wsp* gene).

### *Wolbachia* elimination

We generated a *Wolbachia* uninfected line using antibiotics. Freshly-laid eggs were surface-sterilized in 2.7% sodium hypochlorite (Sigma-Aldrich, St. Louis, MO) for 2 min and twice in 70% ethanol for 2 min. Following two 1 min rinses in distilled water, they were raised on a normal cornmeal, molasses, and yeast food containing 50 µg/mL tetracycline hydrochloride (Wako Pure Chemical Industries, Osaka, Japan). After tetracycline treatment for three generations, a single female was transferred to a vial to establish isofemale lines. After laying eggs, each female was assayed for the presence or absence of *Wolbachia* infections by PCR (see Supplementary Methods). After selection of the vial derived from an uninfected female, flies were maintained on tetracycline-free normal food. Because antibiotic treatment can eliminate other fly microorganisms besides *Wolbachia* such as beneficial gut-associated microbes, these were restored by backcrossing *Wolbachia* uninfected tetracycline-treated females with control infected males as described previously^33^.

### Behavioral analysis

Locomotor activity monitoring was carried out with 3–7-day-old male flies in *Drosophila* activity monitors (DAM2, Trikinetics, Waltham, MA) set in a temperature-controlling incubator (MR-553, Sanyo Electric, Osaka, Japan). Ambient temperature levels (19 °C, 24 °C, or 29 ± 1 °C) and lighting (250 lux, day light fluorescent bulb) were controlled by the incubator. Double-plotting actograms and chi-square periodograms of circadian locomotor rhythms were analyzed using originally developed software written by M.I. (PACR ver. 2.0). To analyze free-running locomotor rhythms, flies were first entrained to 12:12-h LD cycles for 4 days, and then the lighting schedule was switched to DD. For the LD shift experiment, the light phase was lengthened (i.e., phase-delayed) or shortened (i.e., phase-advanced) by 8 h on the 7th day of locomotor activity monitoring under standard LD cycles.

### Immunostaining

Anti-PER immunostaining protocols were described previously^22^. *Wolbachia* were visualized within various adult organs from the *D. melanogaster per-luc* line using an anti-hsp60 antibody (Sigma-Aldrich) which recognizes *Wolbachia*^26–28^. Oocytes, MTs, and brains were dissected from adults in a buffered salt solution containing 137 mM NaCl, 5.4 mM KCl, 0.17 mM NaH_2_PO_4_, 0.22 mM KH_2_PO_4_, 33.3 mM D-glucose, 43.8 mM sucrose, and 10 mM HEPES (pH7.4). Isolated oocytes, MTs, or brains were fixed using 4% (w/v) paraformaldehyde in phosphate-buffered saline (PBS) for 5–15 min at room temperature. Tissues were then blocked in PBS with 10% (v/v) normal goat serum (Vector Laboratories, Burlingame, CA) and 0.1% (v/v) TritonX-100 (Sigma-Aldrich) overnight at 4 °C. Oocytes and MTs were incubated in 1:500 affinity-isolated anti-hsp60 C-terminal antibody (Sigma-Aldrich) for 24 h at 4 °C. Following five 10-min rinses in PBS, samples were incubated with 1:500 Alexa Fluor 488-conjugated goat anti-rabbit IgG [H + L] (Life Technologies, Carlsbad, CA) for 24 h at 4 °C. After five 10-min rinses in PBS, the samples were counter-stained with 1:1,000 DAPI (Dojindo Laboratories, Kumamoto, Japan). Finally, the samples were mounted on glass slides and embedded with 80% glycerol. To identify lateral neurons, brains were incubated with a range of different antibodies with five 10-min rinses in PBS in-between each antibody incubation. The antibodies were: 1:5 mouse monoclonal antibody to *Drosophila* PDF neuropeptide (PDF C7, Developmental Studies Hybridoma Bank, University of Iowa) for 48 h at 4 °C, 1:500 Alexa Fluor 555-conjugated goat anti-mouse IgG [H + L] (Life Technologies) for 24 h at 4 °C, 1:500 anti-hsp60 antibody for 24 h at 4 °C, and 1:500 Alexa Fluor 488-conjugated goat anti-rabbit IgG [H + L] (Life Technologies) for 24 h at 4 °C. After a further five 10-min rinses in PBS, brains were mounted on glass slides and embedded with 80% glycerol. Immunofluorescence images were viewed using a confocal laser-scanning system (A1RMP plus; Nikon, Tokyo, Japan).

### Bioluminescence assay

Flies entrained to a 12:12-h LD cycle were briefly anesthetized with CO_2_ and their heads immediately removed. A single headless body was transferred to a 1.5 mL tube and dipped in Schneider’s *Drosophila* medium (Invitrogen, Carlsbad, CA) supplemented with 20% (v/v) heat-inactivated fetal bovine serum, 500 ng/mL insulin, 100 units/mL penicillin, 100 µg/mL streptomycin, and 1 mM beetle luciferin (Promega, Madison, WI). A one-shot image of bioluminescence was acquired using a deep cooled EM-CCD camera (iXon Ultra-888; Andor Technology, Belfast, UK) with a 5× objective lens (PL-Fluotar 5×/0.15; Leica Microsystems, Wetzlar, Germany). Circadian rhythms in the bioluminescence were analyzed using a temperature-controlled (24 ± 1 °C) luminometer (Turner Designs TD-20/20, Promega). The bioluminescence signals were measured at intervals of 0.2 s and integrated every 3 min.

### Statistical analysis

Means were calculated with standard errors. Student’s *t*-test was used for pairwise comparisons. One-way analysis of variance (ANOVA) followed by Duncan’s multiple range test was used for statistical comparisons among multiple groups. Nuclear PER immunostaining levels from several samples were analyzed using a regression sine curve fitted with 95% confidence levels using SigmaPlot ver 7.0 software (IBM SPSS Statistics, Armonk, NY).

## Electronic supplementary material

Supplementary Information

## Data Availability

DNA and RNA sequences: The nucleotide sequences determined in this study were deposited in the DDBJ/EMBL/GenBank nucleotide sequence database under accession numbers LC108848 to LC108849.
