# Supplementary material for: Nighttime activities and peripheral clock oscillations depend on Wolbachia endosymbionts in flies
Source: Sci Rep. 2018 Oct 18;8:15432. doi: 10.1038/s41598-018-33522-8 (PMC6194088; doi:10.1038/s41598-018-33522-8)
Supplement: Supplementary file 1 — Supplementary Information [file 41598_2018_33522_MOESM1_ESM.docx]

**SupplementaRY Information** for

Nighttime activities and peripheral clock oscillations depend on *Wolbachia* endosymbionts in flies

Eri Morioka, Minami Oida, Tsutomu Tsuchida, and Masayuki Ikeda*

Graduate School of Science and Engineering, University of Toyama

3190 Gofuku, Toyama 930-8555 Japan

***Correspondence:** msikeda@sci.u-toyama.ac.jp

**This PDF file includes:**

Methods

Table S1

Figures S1 to S5

References

**Supplementary Methods**

**Real-time RT-PCR assay**

*wsp* mRNA was analyzed and quantified by referring to a housekeeping gene (*ribosomal protein 49; rp49*) in *per-luc*, tetracycline-treated *per-luc*, *per^01^*, *Clk^Jrk^*, and *cry^01^* adult whole flies or 10 adult brains and 10 adult MTs of infected and uninfected *per-luc* flies using a real-time RT-PCR system (Rotor-Gene Ver. 6 software; Corbett Research, Sydney, Australia). All flies were entrained to 12:12-h LD cycles, and RNA was collected during the light period. Ten individuals (five females and five males) of each fly line were transferred to 1.5 mL RNase-free tubes, and homogenized with a sterile plastic pestle (Funakoshi, Tokyo, Japan) in 350 µL of RLT buffer (RNeasy Mini Kit; Qiagen, Hilden, Germany). Isolated tissues were washed three times in PBS, pooled into 1.5 mL tubes, and then vortex for 10 sec in 350 µL of RLT buffer. Total RNA extraction and reverse transcription procedures were described previously^1^. PCR primer design for *wsp*^2^ and *rp49*^3^ have been described previously. Each primer (1 µM) was used with the Rotor-Gene SYBR Green PCR kit (Qiagen) in the 72-well rotor of the PCR system (Rotor-Gene 3000A; Corbett Research) as described^1^. mRNA levels were expressed using the 2^-ΔΔCt^ method with *rp49* mRNA levels as an internal standard.

**Western blotting**

Flies were entrained in 12:12-h LD cycles for 4 days, and then kept under DD. Infected or uninfected flies were collected every 4 h on the 4th day under DD, and their heads were removed on ice. The headless bodies were immediately frozen at –80°C until use. Protein was extracted from 20 fly bodies per time point in NETN lysis buffer (50 mM Tris-HCl [pH 7.5], 150 mM NaCl, 1 mM EDTA, [pH 8.0], 0.5 mM MgCl_2_, 0.5% Triton X-100, 0.25% NP-40) with a complete protease inhibitor cocktail (Roche, Basel, Switzerland). Samples were homogenized in a 1.5 mL microfuge tube with a plastic pestle (Kimble Chase, Vineland, NJ), temporally sonicated, and then centrifuged (16,000×*g* for 20 min at 4°C). Total proteins in supernatants were denatured by standard sodium dodecyl sulfate buffer (Laemmli Sample Buffer, Bio-Rad, Hercules, CA) with 5% 2-mercaptoethanol (Wako Pure Chemical Industries) and heated at 70°C for 10 min. Equal protein loading was confirmed by gel electrophoresis and Coomassie staining of gels loaded with equal volumes of lysate. Immunoblotting was performed after protein transfer to nitrocellulose membranes. After blocking of membranes using 2% ECL Blocking Agent (GE Healthcare, Amersham, UK) and 0.1% Triton X-100 diluted in PBS, membranes were incubated for 1 h at room temperature with antibody diluted in the same blocking buffer. Following two 5-min rinses in PBS, membranes were incubated with 1:5,000 mouse monoclonal anti-*β*-actin (A2228; Sigma-Aldrich) or 1:10,000 mouse anti-2 Cys Peroxiredoxin antibody [6E5] (AB_16765; Abcam, Cambridge, UK) for 20 h at room temperature. After five 10-min rinses in PBS, membranes were incubated with 1:5,000 Amersham ELC anti-mouse IgG, horseradish peroxidase-linked whole antibody (GE Healthcare). The blot was visualized using chemiluminescence detection.

**Statistical analysis**

Kruskal-Wallis test followed by Steel-Dwass test was used to compare mRNA expression profiles.

**Table S1. Reagent or resource information including their source and identifiers.**

| REAGENT or RESOURCE | SOURCE | IDENTIFIER |
| --- | --- | --- |
| Antibodies | | |
| Rabbit anti-PER | Filgen | Cat#MBS610604 |
| Donkey anti-rabbit IgG - Cyanine Cy3 | Jackson ImmunoResearch | Cat#711-165-152; RRID:AB_2307443 |
| Rabbit anti-hsp60, C-terminal | Sigma-Aldrich | Cat#SAB4501464; RRID:AB_10746162 |
| Goat anti-mouse IgG - Alexa Fluor 555 | Life Technologies | Cat#A-21422; RRID:AB_2435844 |
| Mouse anti-PDF | Developmental Studies Hybridoma Bank | Cat#PDF C7; RRID:AB_760350 |
| Goat anti-rabbit IgG - Alexa Fluor 488 | Life Technologies | Cat#A-11008; RRID:AB_143165 |
| Mouse anti-*β*-actin | Sigma-Aldrich | Cat#A2228; RRID:AB_476697 |
| Mouse anti-2 Cys Peroxiredoxin | Abcam | Cat#ab16765; RRID:AB_443456 |
| Sheep anti-mouse IgG, Whole - Horseradish Peroxidase | GE Healthcare | Cat#NXA931; RRID:AB_772209 |
| Chemicals, Peptides, and Recombinant Proteins | | |
| NaCl | Wako Pure Chemical Industries | Cat#195-01663 |
| Tris-HCl | Sigma-Aldrich | Cat#252859 |
| EDTA | Sigma-Aldrich | Cat#E5513 |
| Ex Taq DNA polymerase | Takara Bio | Cat#PR01AM |
| Sodium hypochlorite | Wako Pure Chemical Industries | Cat#194-02216 |
| Ethanol | Wako Pure Chemical Industries | Cat#057-00456 |
| Tetracycline hydrochloride | Wako Pure Chemical Industries | Cat#209-16561 |
| KCl | Wako Pure Chemical Industries | Cat#163-03545 |
| NaH_2_PO_4_ | Wako Pure Chemical Industries | Cat#192-02815 |
| KH_2_PO_4_ | Nacalai Tesque | Cat#287-21 |
| D-glucose | Wako Pure Chemical Industries | Cat#041-00595 |
| Sucrose | Wako Pure Chemical Industries | Cat#196-00015 |
| HEPES | Wako Pure Chemical Industries | Cat#346-01373 |
| Paraformaldehyde | Wako Pure Chemical Industries | Cat#162-16065 |
| Normal donkey serum | Jackson ImmunoResearch | Cat#017-000-121 |
| Normal goat serum | Vector Laboratories | Cat#S-1000 |
| TritonX-100 | Sigma-Aldrich | Cat#T9284 |
| 4’,6-diamidino-2-phenylindole | Dojindo Laboratories | Cat#340-07971 |
| Glycerol | Wako Pure Chemical Industries | Cat#075-00616 |
| Schneider’s drosophila medium | Invitrogen | Cat#21720-024 |
| Fatal bovine serum | BIOSERA | Cat#515-99055 |
| Insulin | Sigma-Aldrich | Cat#I1882 |
| Penicillin-streptomycin | Invitrogen | Cat#15140-122 |
| Beetle luciferin | Promega | Cat#E1602 |
| MgCl_2 ·_ 6H_2_O | Wako Pure Chemical Industries | Cat#132-00175 |
| 10 % NP-40 | Abcam | Cat#ab142227 |
| cOmplete^TM^ Protease inhibitor cocktail | Roche | Cat#11697498001 |
| Laemmli Sample Buffer | Bio-Rad | Cat#1610737 |
| 2-mercaptoethanol | Wako Pure Chemical Industries | Cat#133-14571 |
| ECL Blocking Agent | GE Healthcare | Cat#RPN2125 |
| Critical Commercial Assays | | |
| Rneasy Mini Kit | Qiagen | Cat#74106 |
| QuantiTect Reverse Transcription Kit | Qiagen | Cat#205311 |
| Rotor-Gene SYBR Green PCR Kit | Qiagen | Cat#204074 |
| Deposited Data | | |
| Nucleotide sequence data of the *Wolbachia* 16S rRNA and *wsp* gene in *D. melanogaster* *per-luc* line. | This paper | DDBJ Accession No. LC108848 – LC108849 |
| Experimental Models: Organisms/Strains | | |
| *D. melanogaster*: *per-luc* | Brandes et al., 1996^4^ | FlyBase: FBtp0006394 |
| *D. melanogaster*: *per^01^* | Konopka and Benzer., 1971^5^ | Flybase: FBal0013649 |
| *D. melanogaster*: *Clk^Jrk^* | Allada et al., 1998^6^ | Flybase: FBal0090722 |
| *D. melanogaster*: *cry^01^* | Dolezelova et al., 2007^7^ | Flybase: FBal0218575 |
| Oligonucleotides | | |
| Primer: 16SA1 Forward: AGAGTTTGATCMTGGCTCAG | Fukatsu and Nikoh 1998^8^ | N/A |
| Primer: 16SB1 Reverse: TACGGYTACCTTGTTACGACTT | Fukatsu and Nikoh 1998^8^ | N/A |
| Primer: wsp Forward: GGGTCCAATAAGTGATGAAGAAAC | Kondo et al., 2002^2^ | N/A |
| Primer: wsp Reverse: TTAAAACGCTACTCCAGCTTCTGC | Kondo et al., 2002^2^ | N/A |
| Primer: rp49 Forward: CTGCCCACCGGATTCAAG | Benito et al., 2008^3^ | N/A |
| Primer: rp49 Reverse: CGATCTCGCCGCAGTAAAC | Benito et al., 2008^3^ | N/A |
| Software and Algorithms | | |
| BLAST | Altschul et al. 1990^9^ | https://blast.ncbi.nlm.nih.gov/Blast.cgi |
| MEGA 5 | Tamura et al., 2011^10^ | https://www.megasoftware.net/ |
| PACR ver. 2.0 |  | N/A |
| Rotor-Gene Ver. 6 | Corbett Research | N/A |
| SigmaPlot ver 7.0 | IBM SPSS Statistics | N/A |

**
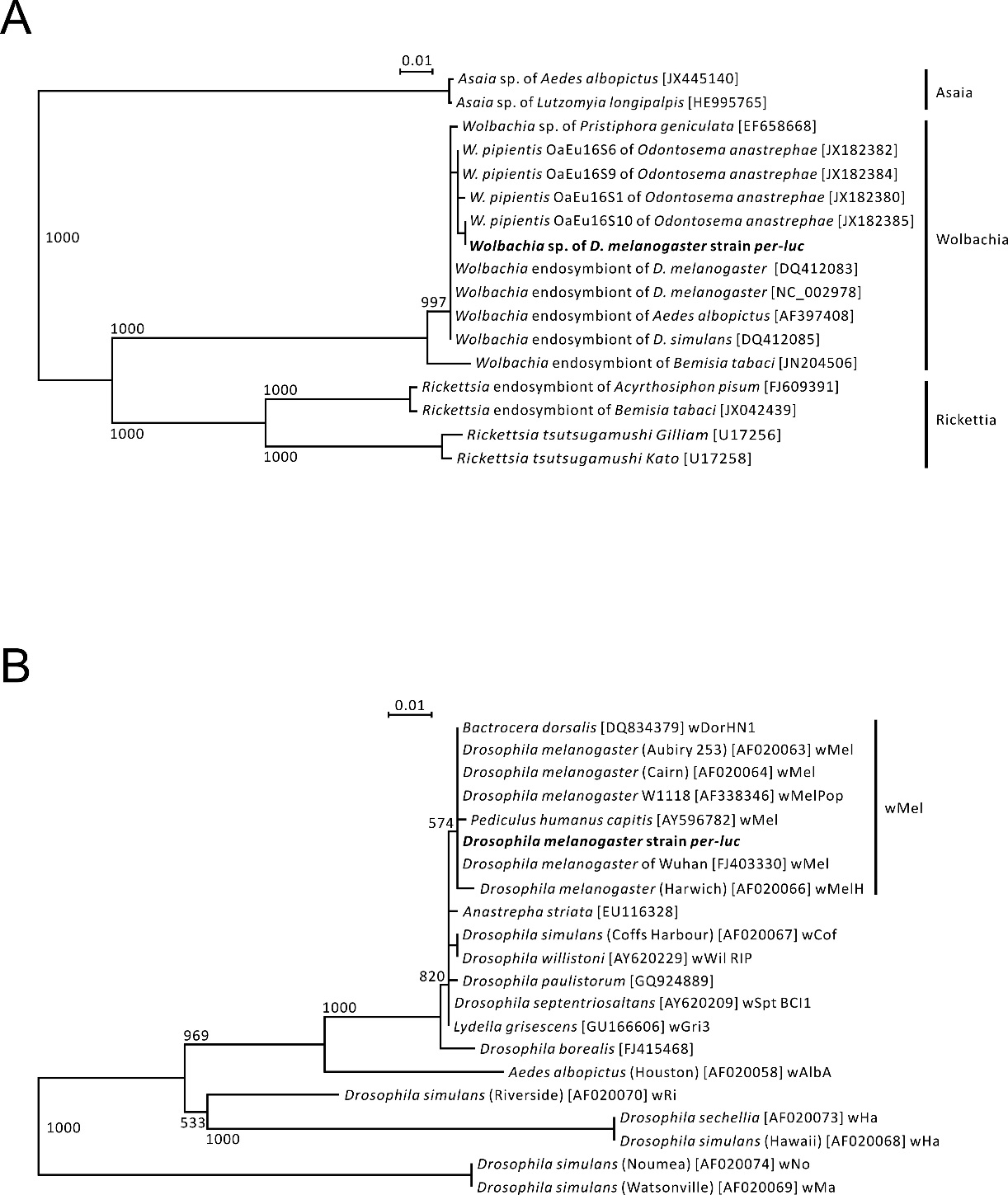
**

**Supplementary Figure S1.** Phylogenetic analysis of *Wolbachia* in *per-luc* strain flies. Phylogenetic trees based on bacterial 16S rRNA (A) and *wsp* (B) sequences using the neighbor-joining method. Numbers above the branches indicate bootstrap support indexes. Scale bars indicate an evolutionary distance of 0.01 nucleotides per position in the sequence. The sequence from this study (*D. melanogaster* strain *per-luc*) is in bold. (A) *Asaia* sp. was used as an outgroup. The name of the bacterium or host insect species is followed by the accession number. (B) The name of the host insect species followed by the accession number and *Wolbachia* strain designation are shown where available.


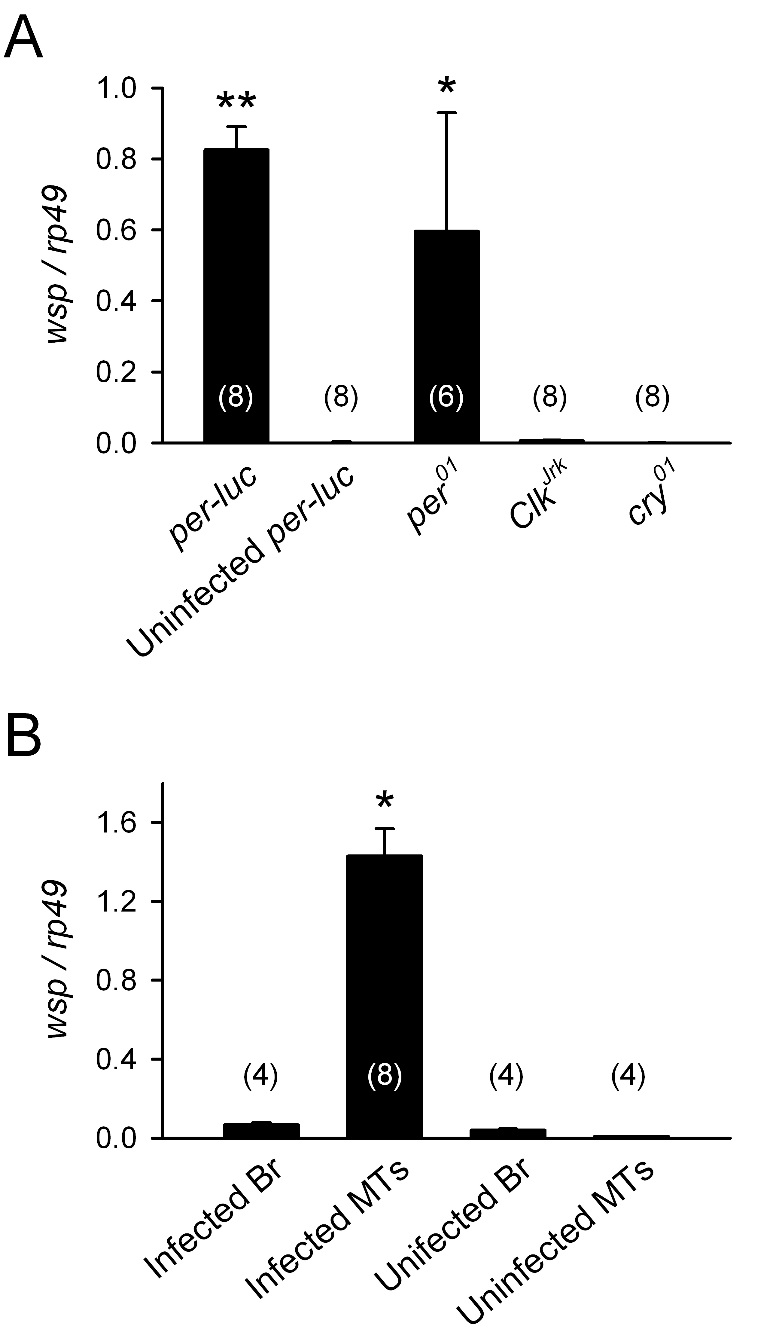


**Supplementary Figure S2.** Real-time RT-PCR assay for genes encoding *wsp*. Relative *wsp* mRNA abundance was quantified using the levels of *rp49* expression. (A) *wsp* expression was detected in *per-luc* and *per^01^* fly strains but not in *Clk^Jrk^* and *cry^01^* flies. *wsp* was also not detected in tetracycline-treated uninfected *per-luc* flies. (B) *wsp* expression was only detected in infected fly MTs but not in infected fly brains (Br), uninfected fly MTs or brains. ***P* < 0.01 and **P* < 0.05 by Kruskal-Wallis test followed by Steel-Dwass test.

**
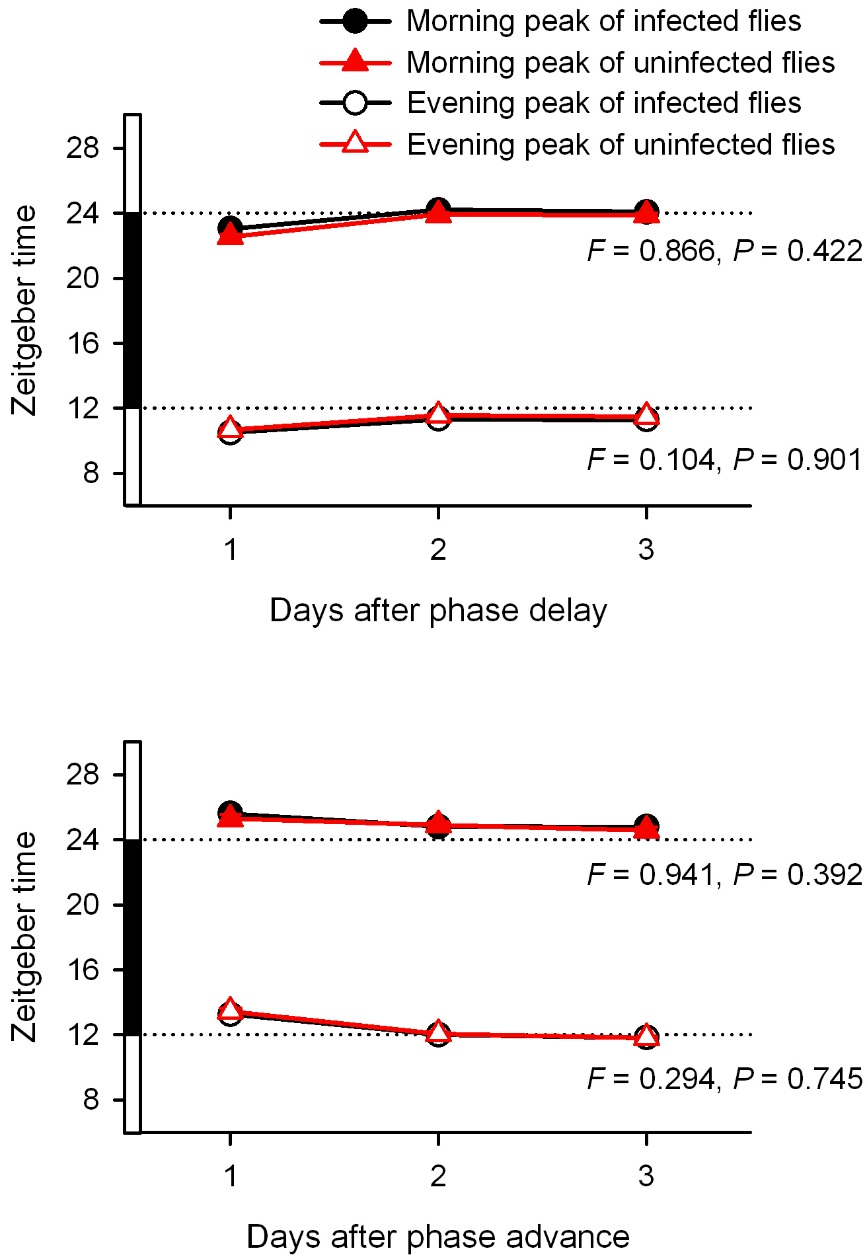
**

**Supplementary Figure S3.** Mean Zeitgeber time of morning peak (closed symbols) and evening peak (open symbols) for 3 days after 8 h phase delay (upper) or advance (lower) of infected (black) and uninfected (red) flies at 24 °C. Data are presented as mean ± SE (n = 17–46). *F* and *P* values mean comparison for interaction effects by two-way ANOVA followed by Tukey’s multiple range test. Examples of rhythms are shown in Figure 3.

**
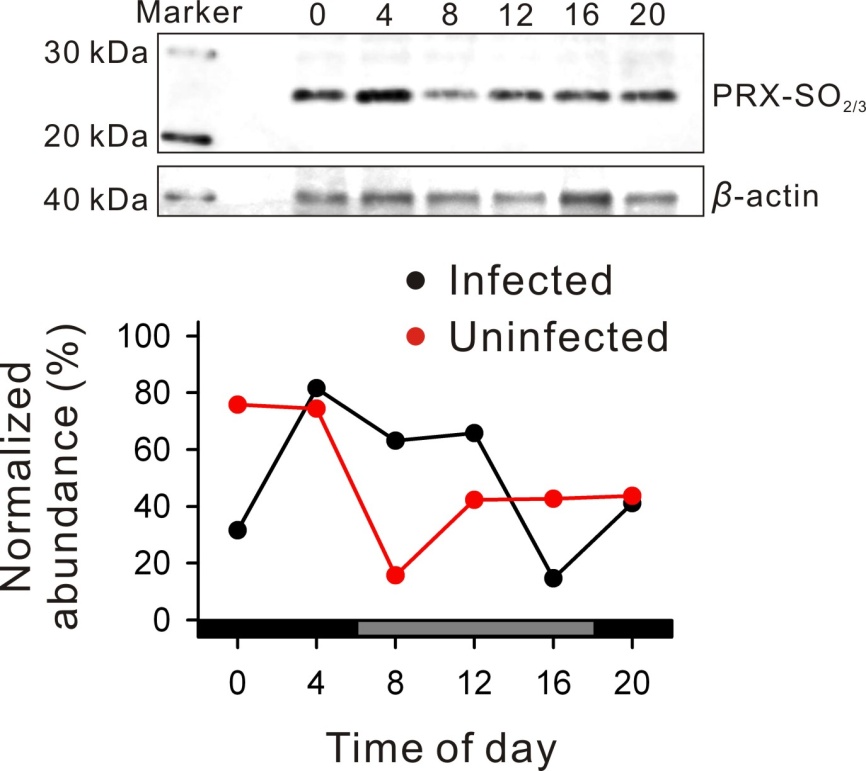
**

**Supplementary Figure S4.** Quantification of oxidized/hyperoxidized 2-Cys peroxiredoxin (PRX-SO_2/3_) in infected (black) and uninfected (red) fly bodies on the 4th day under DD. Normalized abundance was calculated using *β*-actin as a loading control. N = 2–4 for each group. Representative western blotting for uninfected flies is shown on the top.


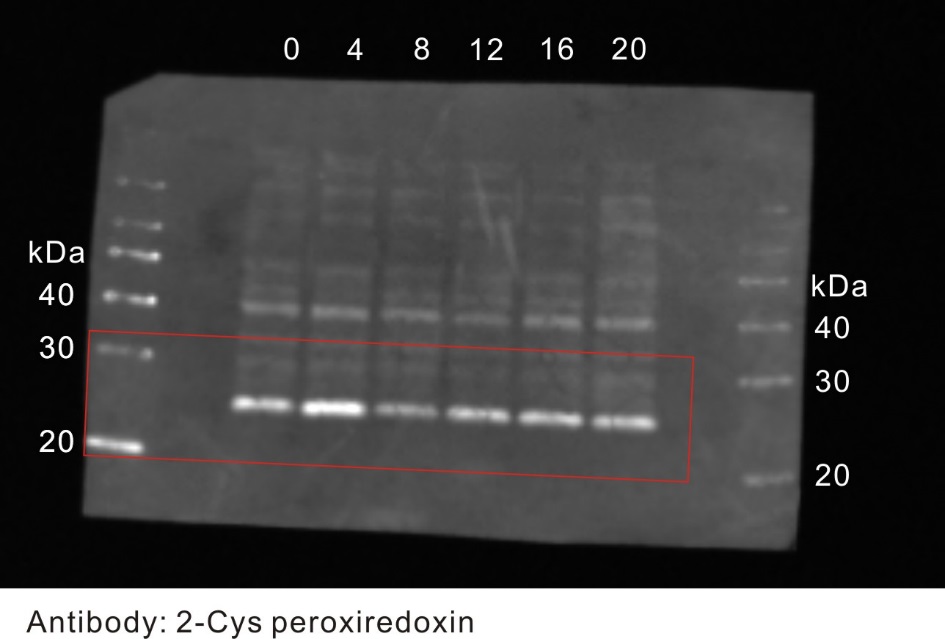


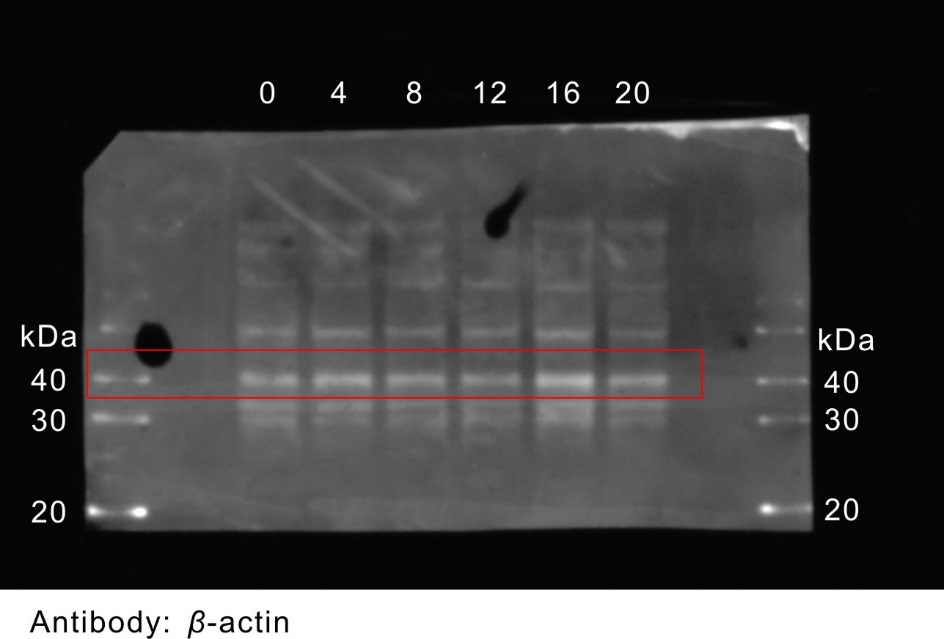


**Supplementary Figure S5.** Chemiluminescent (original) images of western blotting films used for the assay in Supplementary Figure S4.

**References**

1. Morioka, E., Kanda, Y., Koizumi, H., Miyamoto, T. & Ikeda, M. Histamine receptor regulates molecular clock oscillations in human retinal pigment epithelial cells via H_1_ receptors. *Front. Endocrinol. (Lausanne)* **9***,*108; 10.3389/fendo.2018.00108 (2018).
2. Kondo, N., Ijichi, N., Shimada, M. & Fukatsu, T. Prevailing triple infection with *Wolbachia* in *Callosobruchus chinensis* (Coleoptera: Bruchidae). *Mol. Ecol.* **11**, 167-180 (2002).
3. Benito, J., Houl, J. H., Roman, G. W. & Hardin, P. E. The blue-light photoreceptor CRYPTOCHROME is expressed in a subset of circadian oscillator neurons in the *Drosophila* CNS. *J. Biol. Rhythms* **23**, 296-307 (2008).
4. Brandes, C. *et al.* Novel features of Drosophila *period* transcription revealed by real-time luciferase reporting. *Neuron* **16**, 687-692 (1996).
5. Konopka, R. J. & Benzer, S. Clock mutants of *Drosophila melanogaster*. *Proc. Natl. Acad. Sci. USA* **68**, 2112-2116 (1971).
6. Allada, R., White, N. E., So, W. V., Hall, J. C. & Rosbash, M. A mutant *Drosophila* homolog of mammalian *Clock* disrupts circadian rhythms and transcription of *period* and *timeless*. *Cell* **93**, 791-804 (1998).
7. Dolezelova, E., Dolezel, D. & Hall, J. C. Rhythm defects caused by newly engineered null mutations in Drosophila's *cryptochrome* gene. *Genetics* **177**, 329-345 (2007).
8. Fukatsu, T. & Nikoh, N. Two intracellular symbiotic bacteria from the mulberry psyllid *Anomoneura mori* (Insecta, Homoptera). *Appl. Environ. Microbiol.* **64**, 3599-3606 (1998).
9. Altschul, S. F., Gish, W., Miller, W., Myers, E. W. & Lipman, D. J. Basic local alignment search tool. *J. Mol. Biol.* **215**, 403-410 (1990).
10. Tamura, K. *et al.* MEGA5: molecular evolutionary genetics analysis using maximum likelihood, evolutionary distance, and maximum parsimony methods. *Mol. Biol. Evol.* **28**, 2731-2739 (2011).
